# Differences in Survivability under Starvation Conditions Among Four Species of Purple Nonsulfur Phototrophic Bacteria

**DOI:** 10.1264/jsme2.ME14013

**Published:** 2014-06-18

**Authors:** Nanako Kanno, Katsumi Matsuura, Shin Haruta

**Affiliations:** 1 Graduate School of Science and Engineering, Tokyo Metropolitan University, Minami-Osawa 1–1, Hachioji, Tokyo 192–0397, Japan

**Keywords:** starvation, survivability, purple phototrophic bacteria

## Abstract

Survivability under carbon-starvation conditions was investigated in four species of purple phototrophic bacteria: *Rhodopseudomonas palustris*, *Rhodobacter sphaeroides*, *Rhodospirillum rubrum*, and *Rubrivivax gelatinosus*. All these test organisms survived longer in the light than in the dark. ATP levels in the cultures were maintained in the light, which indicated that survivability was supported by photosynthesis. Survivability and tolerance against hypertonic stress in the dark was higher in *Rhodopseudomonas palustris*, which is widely distributed in natural environments including soils, than in the three other species.

Purple nonsulfur phototrophic bacteria grow by anoxygenic photosynthesis and utilize organic carbon as a carbon source for growth. They have been shown to markedly contribute to carbon, nitrogen, and sulfur cycles on the Earth (10). Previous studies examined the survivability of purple phototrophic bacteria under starvation conditions (2, 11). Breznak *et al.* (2) reported that the carbon-starved cells of *Rhodospirillum rubrum* strain Ha remained viable for longer in the light than in the dark. Oda *et al.* (11) demonstrated the positive effects of illumination on the survivability of *Rhodopseudomonas palustris* strain DCP3. The positive effects of light on starvation survival have also been reported in some strains of aerobic bacteriochlorophyll-containing bacteria (4, 7, 13, 14). Energy supply by photosynthesis appears to promote survivability; however, no direct correlation between the energy level of cells and viability has not yet been reported.

In the present study, we determined the viability and ATP levels of the test organisms under carbon-starvation conditions in the light and dark. Four species were used comparatively; *Rhodopseudomonas palustris* strain ATCC BAA-98 (=CGA009), *Rhodobacter sphaeroides* strain ATCC 17023^T^ (=2.4.1^T^), *Rhodospirillum rubrum* strain S1^T^ (=ATCC 11170^T^), and *Rubrivivax gelatinosus* strain IL144 (=NBRC 100245). The susceptibility of the starved cells of these species to osmotic stress was also investigated in order to characterize their survivability.

*Rps. palustris* ATCC BAA-98 and *Rba. sphaeroides* ATCC 17023^T^ were obtained from the American Type Culture Collection and *Rsp. rubrum* S1^T^ and *Rvi. gelatinosus* IL144 (=NBRC 100245) were kindly provided by K. Shimada (Tokyo Metropolitan University). These bacteria were anaerobically cultivated in a carbon-limited medium (pH 7.0) containing (per liter) 0.5 g sodium succinate as the sole source of carbon, 1 g (NH_4_)_2_SO_4_, 0.38 g KH_2_PO_4_, 0.39 g K_2_HPO_4_, 1 mL of a vitamin mixture (5), and 5 mL of a basal salt solution (5) at 30°C under illumination (tungsten lamp with a 750 nm longpass filter; 600 J s^−1^ m^−2^, quantitated by a pyranometer [LI-190SA, Meiwafosis, Tokyo, Japan]). The cultures were continuously and vigorously agitated using a magnetic stirrer. When increases in optical density at 660 nm ceased at the exponential growth phase, the culture was defined as being in carbon-starvation conditions. The starved cells in vials were incubated at 30°C with agitation in the light as described above or in the dark. Viability was measured by plate counting with 1.5% agar medium containing (per liter) 1 g sodium succinate, 1 g sodium acetate, 0.1 g yeast extract (Nihon Seiyaku, Tokyo, Japan), 0.1 g Na_2_S_2_O_3_·5H_2_O, 1 g (NH_4_)_2_SO_4_, 0.38 g KH_2_PO_4_, 0.39 g K_2_HPO_4_, 1 mL of a vitamin mixture (5), and 5 mL of a basal salt solution (5). To determine CFUs, serial dilutions of cultures were spread on 2 to 4 agar plates per one of the serial dilutions and cultivated aerobically at 30°C in the dark for approximately one week. The amount of ATP in the cultures was quantified using the BacTiter-Glo Microbial Cell Viability Assay Kit (Promega, Madison, WI, USA) and GloMax 20/20n Luminometer (Promega) according to the manufacturer’s protocol.

The four test strains of phototrophic bacteria were grown photoheterotrophically (Fig. S1). When sufficient organic carbon was available (5 g sodium succinate per liter), the cultures reached the stationary phase at which the optical densities at 660 nm were 0.5–1.6. This retardation of growth did not appear to be due to carbon starvation, but may have been attributed to a high cell density. When the initial concentration of sodium succinate as the carbon source was reduced to 0.5 g per liter, growth stopped at an optical density of 0.2–0.3 (Fig. S1); these cells were then defined as carbon-starved cells. Although the production of fumarate was observed in the culture of *Rps. palustris* ATCC BAA-98 during the exponential phase of growth, neither succinate nor fumarate were detected in the culture supernatant after growth stopped under carbon-limiting conditions (data not shown). Viability and ATP levels were determined in the starved cultures of *Rps. palustris* ATCC BAA-98 and *Rsp. rubrum* S1^T^ after incubation in the light and dark (Fig. 1). In both strains, higher CFU values were observed in the light than in the dark. This higher CFU-value in the light was also confirmed for *Rba. sphaeroides* ATCC 17023^T^ and *Rvi. gelatinosus* IL144 (Fig. S2). ATP levels were also maintained in the light (Fig. 1). The ATP produced through cyclic photophosphorylation may be consumed to keep viability by maintaining cytoplasmic homeostasis and/or synthesizing mending proteins. Our preliminary transcriptome analysis of *Rps. palustris* ATCC BAA-98 indicated that the genes related to transcription, translation, and protein decomposition were highly expressed under starvation conditions in the light (data not shown), which suggested that the decomposition and synthesis of cellular proteins may have been stimulated. A previous proteomic study for an aerobic phototrophic bacterium also demonstrated that light induced marked changes in the composition of proteins in cells under carbon-limiting conditions (18).

During the period of incubation in the dark, the viable counts of the four strains of the phototrophic bacteria decreased at different rates from each other. After 10 d from the beginning of starvation, the CFUs of *Rps. palustris* ATCC BAA-98 decreased to 51% of the initial value, while the ATP level decreased more to 7%. When the starved cells that had been maintained in the dark for 10 d were illuminated for 1 min, ATP levels increased by more than 10-fold (data not shown). The decrease of up to 10% in the ATP concentrations in cells under growing conditions may not have been fatal to survival. The CFU and ATP levels of *Rsp. rubrum* S1^T^ rapidly decreased in the dark and reached 0.007% and 0.3% of the initial values after 6 d of starvation, respectively (Fig. 1). This rapid decrease in ATP in *Rsp. rubrum* S1^T^ may have been caused by the excessive consumption of ATP for cellular activities such as swimming motility, but not for maintenance of viability (8).

Susceptibility to osmotic stress was determined using the starved cells (Fig. 2). After exposure to 2.0 M sucrose solution, *Rps. palustris* ATCC BAA-98 and *Rba. sphaeroides* ATCC 17023^T^ maintained high viabilities (Fig. 2a). In contrast, the sucrose stress markedly decreased the viabilities of *Rsp. rubrum* S1^T^ and *Rvi. gelatinosus* IL144. Resistance to the sucrose stress was similar to survivability in the dark among the four species (Figs. 1, 2a and S2). Tolerance to osmotic stress may be achieved by membrane translocators and the composition of membrane fatty acids (1, 15–17). Although it is unlikely given the carbon-starvation conditions used in this study, it may be possible that an osmolyte such as trehalose accumulated in cells (15, 17).

Osmotic stress with NaCl did not largely affect the viability of *Rps. palustris* ATCC BAA-98, whereas the other test organisms were susceptible to this stress (Fig. 2b). It is noteworthy that 15% of the genome in *Rps. palustris* ATCC BAA-98 is relevant to transport including sodium efflux systems (9). This may partially explain *Rps. palustris* being detected in a wide variety of environments such as paddy soil, freshwater marsh sediments, and aquatic sediments (3, 6, 12).

In summary, all four purple phototrophic bacteria used in this study exhibited long-term starvation-survival in the light. This result indicates that they effectively survive in natural environments in which light energy is available even if organic carbon sources are depleted. A physiological characterization of the non-growing cells of phototrophic bacteria under starved conditions will clarify how ATP is utilized to maintain viability.

## Supplementary Information



## Figures and Tables

**Fig. 1:**
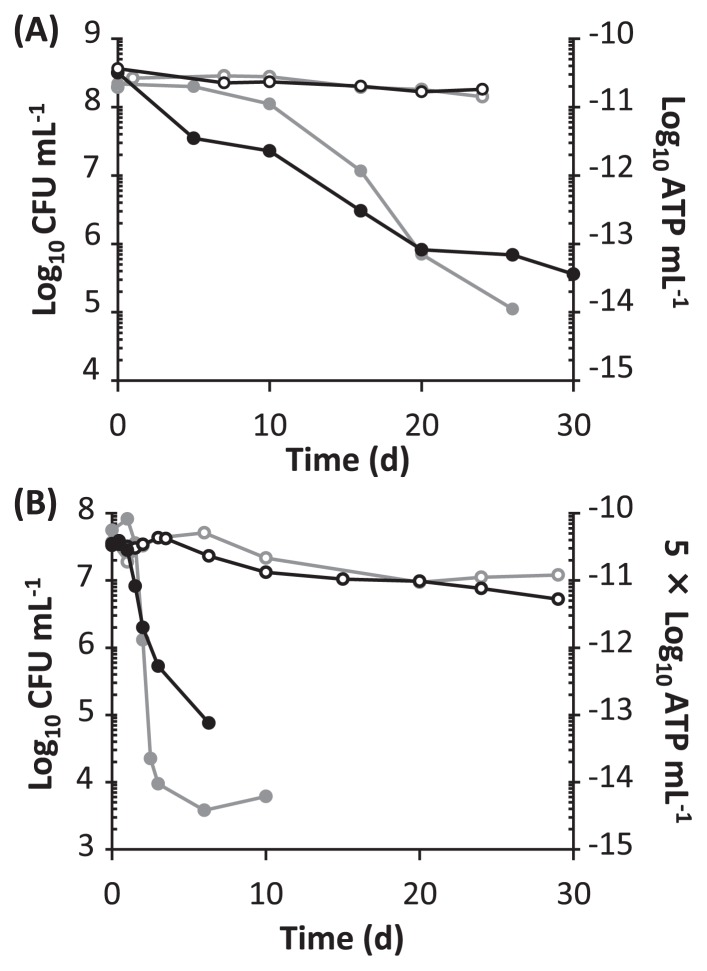
Changes in viability (black line) and ATP levels (gray line) during carbon-starvation conditions. The starved cells of *Rps. palustris* ATCC BAA-98 (A) and *Rsp. rubrum* S1^T^ (B) were incubated in the light (open circle) and dark (filled circle). Time 0 was defined as the time when growth completely stopped. Viability was measured by plate counting and expressed as CFU mL^−1^.

**Fig. 2:**
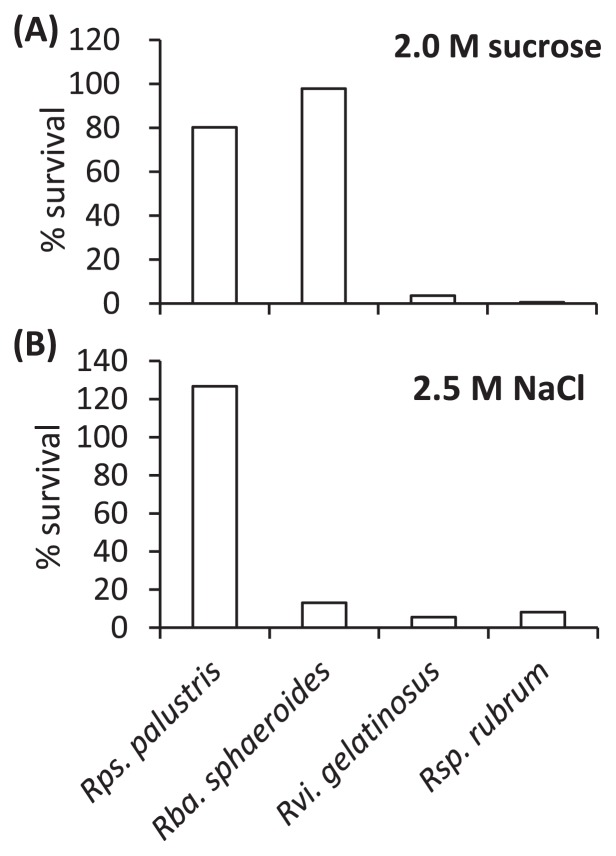
Effects of osmotic stress on the viability of starved cells. Starved cells were suspended in a buffer solution containing (per liter) 0.38 g KH_2_PO_4_, 0.39 g K_2_HPO_4_, 1 mL of a vitamin mixture (5), and 5 mL of a basal salt solution (5), and either 2.0 M sucrose (A) or 2.5 M NaCl (B). CFUs were counted after incubation at 30°C for 10 min in the dark. The results are expressed as a percentage of the CFUs determined after incubation for 10 min without exposure to stress.
