# Differences in Survivability under Starvation Conditions Among Four Species of Purple Nonsulfur Phototrophic Bacteria

**DOI:** 10.1264/jsme2.ME14013e

**Published:** 2017-03

**Authors:** Nanako Kanno, Katsumi Matsuura, Shin Haruta

**Affiliations:** 1 Graduate School of Science and Engineering, Tokyo Metropolitan UniversityMinami-Osawa 1–1, Hachioji, Tokyo 192–0397Japan

Volume 29, no. 3, Page 326–328, 2014

Page 327, Legend for Fig. 1

Incorrect

**Fig. 1.** Changes in viability (black line) and ATP levels (gray line) during carbon-starvation conditions.

Correct

**Fig. 1.** Changes in viability (gray line) and ATP levels (black line) during carbon-starvation conditions.

