# Supplementary material for: Differences in Survivability under Starvation Conditions Among Four Species of Purple Nonsulfur Phototrophic Bacteria
Source: Microbes Environ. 2014 Jun 18;29(3):326–8. doi: 10.1264/jsme2.ME14013 (PMC4159045; doi:10.1264/jsme2.ME14013)
Supplement: Supplementary file 1 [file 29_326_s1.pdf]

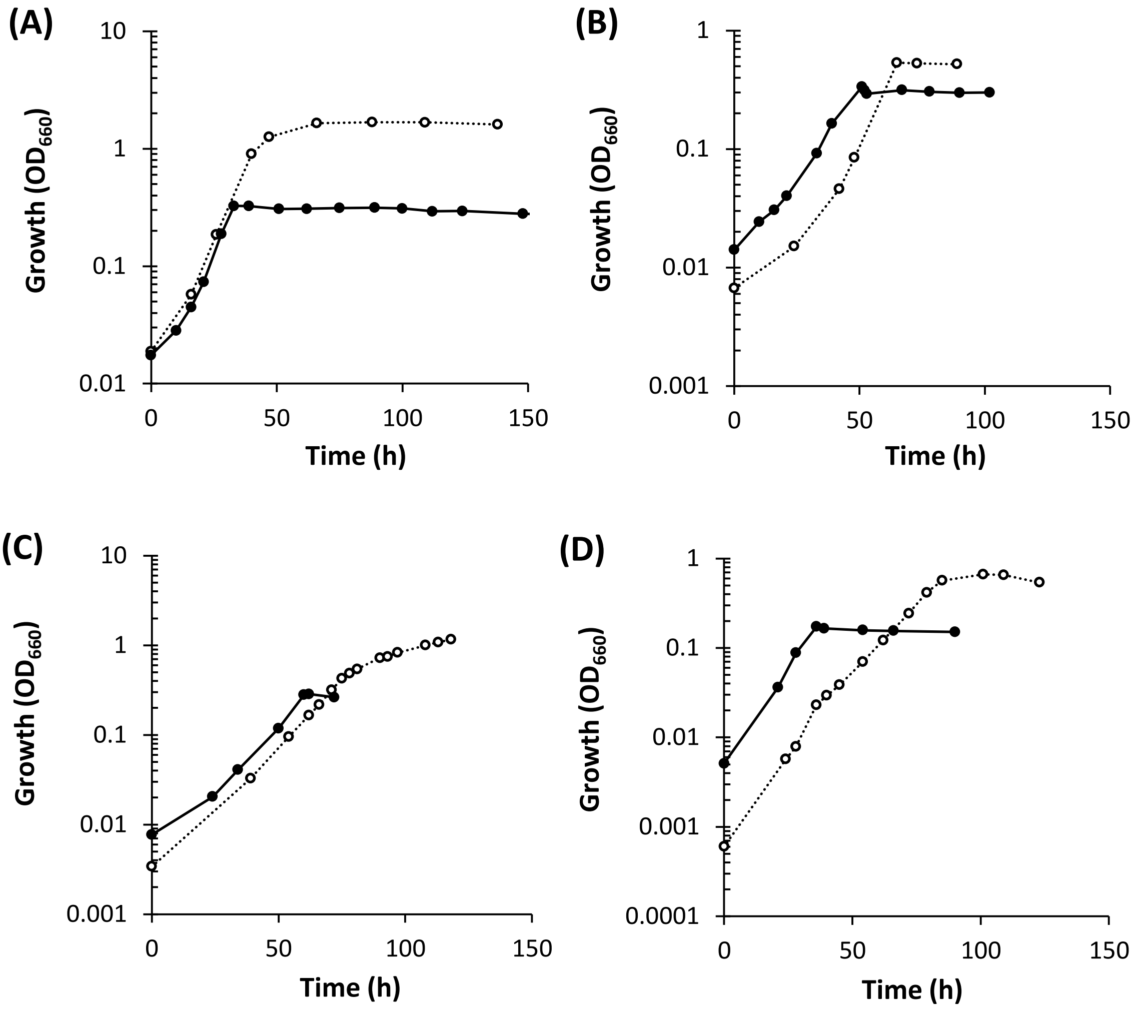

Fig. S1. Growth of the purple nonsulfur phototrophic bacteria in the carbon-limited medium containing 0.5 g sodium succinate per liter (filled circle) or in the carbon-sufficient medium containing 5 g sodium succinate per litter (open circle). The cells of *Rps. palustris* ATCC BAA-98 (A), *Rsp. rubrum* S1^{T} (B), *Rba. sphaeroides* ATCC 17023^{T} (C) and *Rvi. gelatinosus* IL144 (D) were grown under anaerobic light conditions.

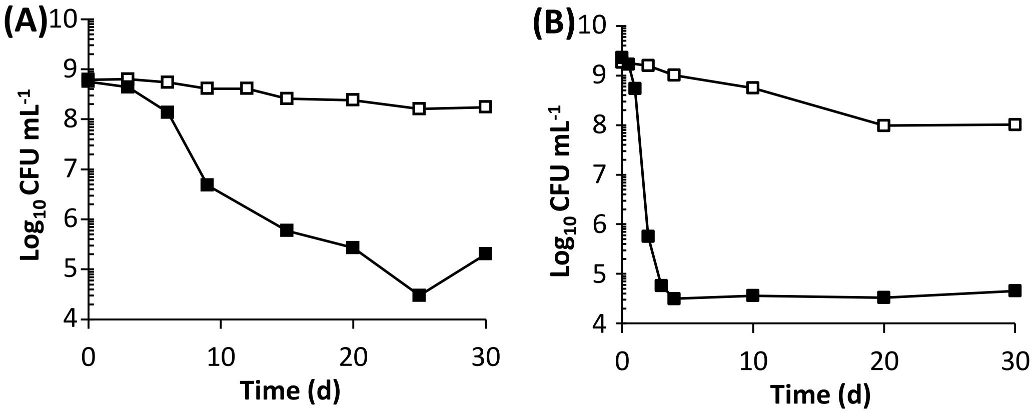

Fig. S2. Change in viability during carbon-starvation conditions. Starved cells of *Rba. sphaeroides* ATCC 17023^T (A) and *Rvi. gelatinosus* IL144 (B) were incubated in the light (open square) and dark (filled square). Time 0 was defined as the time when the growth completely stopped. Viability was measured as described in Fig. 1.
